# Serum Free Fatty Acid-Based Diagnostic Index for Cervical Cancer Including High-Risk HPV-Negative Cases: An Independent Validation Study

**DOI:** 10.3390/diagnostics16203387

**Published:** 2026-10-18

**Authors:** Yuki Katoh, Yosuke Fujii, Nagi Yamazaki, Akiko Kubo, Saki Kamata, Yuki Okuma, Ting Yang, Ayase Futatsugi, Masaki Sakamoto, Hirotsugu Hino, Tadashi Ogawa, Mamiko Fukuta, Takashi Iwata, Hiroshi Nishio, Masaki Sugawara, Kei Kawana, Shuichi Hirai

**Affiliations:** 1 Division of Anatomical Science, Department of Functional Morphology, Nihon University School of Medicine, 30-1 Ohyaguchi-Kami-Cho, Itabashi-ku, Tokyo 173-8610, Japan; fujii.yosuke@nihon-u.ac.jp (Y.F.); mena24120@g.nihon-u.ac.jp (N.Y.); yang.ting@nihon-u.ac.jp (T.Y.); futatsugi.ayase@nihon-u.ac.jp (A.F.); sakamoto.masaki@nihon-u.ac.jp (M.S.); hino.hirotsugu@sapmed.ac.jp (H.H.); hirai.shuichi@nihon-u.ac.jp (S.H.); 2 Department of Obstetrics and Gynecology, Keio University School of Medicine, 35 Shinano-machi, Shinjuku-ku, Tokyo 160-8582, Japan; iwata.a8@keio.jp (T.I.); nishio.z3@keio.jp (H.N.); masaki.sugawara@keio.jp (M.S.); 3 Department of Medicine, Nihon University School of Medicine, 30-1 Ohyaguchi-Kami-Cho, Itabashi-ku, Tokyo 173-8610, Japan; 4 Division of Dermatology, Department of Internal Related Kobe University Graduate School of Medicine, 7-5-1 Kusunoki-Cho, Chuo-ku, Kobe, Hyogo 650-0017, Japan; yuba@med.kobe-u.ac.jp; 5 Department of Obstetrics and Gynecology, Nihon University School of Medicine, 30-1 Ohyaguchi-Kami-Cho, Itabashi-ku, Tokyo 173-8610, Japan; kamata.saki@nihon-u.ac.jp (S.K.); okuma.yuki@nihon-u.ac.jp (Y.O.); kawana.kei@nihon-u.ac.jp (K.K.); 6 Department of Legal Medicine, Aichi Medical University School of Medicine, 1-1 Yazakokarimata, Nagakute, Aichi 480-1195, Japan; ogawatd@aichi-med-u.ac.jp (T.O.); mfukuta@aichi-med-u.ac.jp (M.F.)

**Keywords:** HPV-negative cervical cancer, stearic acid, dihomo-γ-linolenic acid, diagnostic biomarker, liquid biopsy

## Abstract

**Background/Objectives:** Cervical cancer (CC) is usually caused by high-risk human papillomavirus (hrHPV); however, some hrHPV-negative cases are undetectable by current screening programs. We evaluated a previously established diagnostic model based on serum free fatty acids (FFAs) in an independent cohort including hrHPV-negative CC. **Methods:** We analyzed serum FFAs from 101 patients with CC (16 hrHPV-negative), 60 healthy donors, and 16 patients with benign gynecological diseases, none of whom were included in the previous study, and gene expression levels of fatty acid metabolism-related enzymes in 64 surgical CC and five normal cervix samples. **Results:** Levels of nine FFAs differed between CC and the non-cancer groups but not between hrHPV-negative and hrHPV-positive CC. Gene expression levels of acetyl-CoA carboxylase 1, fatty acid synthase, stearoyl-CoA desaturase 1, and monoacylglycerol lipase were increased in CC compared with normal tissues, with no significant differences between hrHPV-negative and hrHPV-positive CC. The previous model (diagnostic index = −5.631 + 0.030 × (stearic acid) + 9.188 × (dihomo-γ-linolenic acid)) distinguished CC from the non-cancer groups (area under the curve, 0.969). At the pre-specified cut-off value, sensitivity was 97.0% (hrHPV-negative CC, 93.8%) and specificity in healthy donors was 58.3%; at the cut-off value derived in this cohort, these were 82.2% (93.8%) and 98.3%, respectively. In hrHPV-negative CC, the positivity rate exceeded those of squamous cell carcinoma antigen, carcinoembryonic antigen, and CA125. **Conclusions:** The serum-FFA-based diagnostic model detected CC, including hrHPV-negative cases, in an independent cohort. With calibration of the cut-off value, this approach may complement existing HPV-based testing.

## 1. Introduction

Cervical cancer (CC) remains one of the main malignancies affecting women worldwide, with approximately 600,000 new cases and 340,000 deaths annually [1,2]. Human papillomavirus (HPV) infection is widely recognized as the principal risk factor for CC, with persistent infection by high-risk HPV (hrHPV) types playing a crucial role in carcinogenesis [3,4]. Based on this understanding, current CC screening programs utilizing a combination of cytology and HPV testing have contributed to the early detection and treatment of CC [5,6,7].

Not all CCs, however, are attributable to hrHPV infection, and current epidemiological data indicate that approximately 5–10% of cases test negative for hrHPV, depending on the population, testing method, and diagnostic criteria [8,9]. These hrHPV-negative CCs are believed to develop through hrHPV-independent carcinogenic mechanisms and have generally been associated with a poorer prognosis [10,11,12]. Gastric-type adenocarcinoma, the most common HPV-independent subtype, frequently harbors alterations in *TP53* and *STK11* [13,14]. Current screening strategies, which are predominantly based on hrHPV testing, may miss these cases, presenting a major clinical issue. There is thus an urgent need to develop new diagnostic approaches capable of effectively detecting hrHPV-negative CC.

Cancer cells have the ability to modify their metabolic pathways to enhance their own survival [15]. Numerous investigations have examined the distinctive glucose and amino acid metabolism pathways in cancer cells, leading to therapeutic and diagnostic applications [16,17,18]. In CC, lipid metabolic reprogramming has been implicated in tumor progression [19]. Fatty acid metabolic reprogramming can be driven by different oncogenic pathways. In HPV-associated CC, inactivation of the retinoblastoma protein and the resulting induction of E2F1 have been reported to promote de novo lipid synthesis [20], whereas in HPV-independent gastric-type adenocarcinoma, frequent alterations in STK11 (LKB1) [13,14] may reduce AMP-activated protein kinase activity and release acetyl-CoA carboxylase from inhibition, thereby enhancing fatty acid synthesis [21]. Fatty acid metabolic reprogramming may therefore occur in CC regardless of whether carcinogenesis is driven by HPV.

We previously demonstrated that colorectal, lung, and ovarian cancers could modify their fatty acid metabolic mechanisms [22,23]. Furthermore, we reported that fatty acid metabolic reprogramming mechanisms also exist in CC, with dramatic changes in serum levels of free fatty acids (FFAs), particularly stearic acid and dihomo-γ-linolenic acid, and we accordingly developed a CC diagnostic model using these two FFAs, which were selected by multivariable analysis [24]. The ability of this diagnostic model to detect hrHPV-negative CC, however, remains unknown.

This study aimed to determine, in an independent cohort that did not overlap with our previous study [24], whether this diagnostic method based on serum-FFA profiles could also detect hrHPV-negative CC. Specifically, we evaluated the diagnostic performance of fatty acid biomarkers in hrHPV-negative CC, focusing on stearic acid and dihomo-γ-linolenic acid, and examined their potential application for the detection of cases that might be missed by current HPV testing. This approach may lead to the development of a CC screening method that complements HPV testing.

## 2. Materials and Methods

### 2.1. Clinical Samples

Serum samples were obtained from 101 patients with CC, 16 patients with benign gynecological diseases, and 60 healthy donors at Nihon University (Tokyo, Japan) and Keio University (Tokyo, Japan) between 2022 and 2025 (Table 1). All samples were stored at −150 °C until use. Clinicopathological information was collected from clinical records and pathology reports. Patients with CC were classified as stage I to stage IV according to the International Federation of Gynecology and Obstetrics (FIGO) 2018 staging system, incorporating pathological findings [25]. Patients were enrolled consecutively, and serum samples were collected from consenting patients before removal of the tumor at the time of initial surgery. The inclusion criteria were histologically confirmed CC and primary surgical treatment. Patients who had received neoadjuvant chemotherapy or any other prior treatment were excluded. Patients whose final histopathological diagnosis differed from the preoperative diagnosis were also excluded. Patients with benign gynecological diseases (endometriosis or uterine fibroids) who underwent total hysterectomy were included as an additional control group, and their serum samples were collected under the same perioperative conditions as those of the patients with CC. None of the participants in this study were included in our previous study [24]. Serum samples from healthy donors were residual samples collected at Nihon University from 2020 onwards in our previous clinical studies of other cancers, such as lung, oral, and pancreatic cancers; samples used in our previous study [24] were not included. Written informed consent for the use of clinical materials for research purposes was obtained from all patients. CC and normal cervical tissue samples were surgically resected from patients at Nihon University and Keio University (Tokyo, Japan). Normal cervical tissues were obtained from macroscopically normal areas of the cervix in patients who underwent total hysterectomy for benign gynecological diseases, such as uterine fibroids.

hrHPV status was determined using the cobas HPV test on the cobas 6800/8800 Systems (Roche Diagnostics, Basel, Switzerland), performed by SRL, Inc. (Tokyo, Japan). Cervical scraping specimens were collected before treatment in ThinPrep PreservCyt solution (Hologic, Marlborough, MA, USA). The assay detects HPV16 and HPV18 individually and 12 other hrHPV genotypes (31, 33, 35, 39, 45, 51, 52, 56, 58, 59, 66, and 68) as a pooled result. Patients negative for all 14 genotypes were classified as hrHPV-negative.

### 2.2. Measurement of Serum FFAs by Gas Chromatography–Mass Spectrometry (GC-MS)

Phosphate-buffered saline (0.3 mL) containing internal standards (100 ng of margaric acid) was added to 20 µL of serum and mixed by vortexing. FFAs were then extracted from samples using an ISOLUTE SLE+ column (Biotage, Vimpelgatan, Uppsala, Sweden) and dichloromethane. The lower organic region of the sample was collected and dried under a nitrogen stream. The residue was dissolved in 5 µL of pyridine and 30 µL of the reagent BSTFA+TMCS (99:1) (TS-38831; Thermo Fisher Scientific, Waltham, MA, USA) for trimethylsilylation. The derivatization reaction was performed for 30 min at 40 °C.

GC-MS analysis was performed on a Shimadzu (Shimadzu Corporation, Kyoto, Japan) GC-MS QP2010 Ultra equipped with an AOC20i autoinjector and Rtx-5MS column (30 m, 0.25 mm, 0.25 µm df) in 70 eV electron ionization mode. The oven temperature program was as follows: 150 °C for 1 min, 20 °C/min to 250 °C, 5 °C/min to 280 °C, hold for 5 min, then 20 °C/min to 330 °C and hold for 3 min, after which the temperature was maintained for 10 min. Helium was used as the carrier gas with a constant flow speed of 42.0 cm/s, with 1 µL injected at a 5:1 split ratio with an injector temperature of 250 °C. The MS interface temperature was held at 280 °C. The target ion (*m*/*z*), retention time, limit of detection, and limit of quantification for the nine FFAs evaluated are summarized in Table S1. FFAs were quantified using external calibration curves of authentic standards, which were constructed for each analytical run over concentration ranges covering those of the samples (r^2^ ≥ 0.97 in all runs). Samples were analyzed in random order, and analysts were blinded to case status. Serum samples from patients with CC and healthy donors were analyzed during the same period, whereas those from patients with benign gynecological diseases were analyzed later in a separate analytical batch.

### 2.3. Quantitative Reverse Transcription-Polymerase Chain Reaction (qRT-PCR) Analysis

CC and normal cervical tissues were immersed in RNAlater (Thermo Fisher Scientific, Waltham, MA, USA) immediately after resection and stored at −80 °C until RNA extraction. Total RNA was extracted from CC and normal cervical tissues using an RNeasy Mini Kit (Qiagen, Hilden, Germany). TaqMan RT-PCR primers and probes for human acetyl-CoA carboxylase 1 (*ACACA*), fatty acid synthase (*FASN*), stearoyl-CoA desaturase 1 (*SCD1*), and monoacylglycerol lipase (*MGLL*) were purchased from Integrated DNA Technologies (Coralville, IA, USA). qRT-PCR was performed using a ReverTra Ace^®^ qPCR RT Kit (Toyobo, Osaka, Japan). Gene expression was determined by the 2−ΔCt method, in which ΔCt is the difference between the mean Ct value of duplicate measurements of the sample and the endogenous 18S rRNA control.

### 2.4. Statistical Analysis

Unsupervised clustering and heatmap generation with sorted datasets were performed using MetaboAnalyst 6.0. Partial least squares discriminant analysis (PLS-DA) was performed using MetaboAnalyst 6.0 for three groups (healthy donors, hrHPV-positive CC, and hrHPV-negative CC), and the model was evaluated by 10-fold cross-validation and a permutation test with 1000 permutations. Differences between two groups were assessed using two-tailed Mann–Whitney U tests, and differences among three or more groups were assessed using the Kruskal–Wallis test followed by Dunn’s multiple comparison test. For comparisons between hrHPV-negative and hrHPV-positive CC, effect sizes were estimated as Hodges–Lehmann differences with 95% confidence intervals (CIs); FFA concentrations and gene expression levels were log2-transformed for this purpose, and effect sizes are expressed as fold changes. *p* values from the Kruskal–Wallis test for the nine FFAs and from the Mann–Whitney U test for the four genes and for comparisons between hrHPV-negative and hrHPV-positive CC were adjusted using the Benjamini–Hochberg false discovery rate (FDR) method. The diagnostic sensitivity, specificity, and area under the curve (AUC) values were calculated for each FFA. The diagnostic index (DI) (stearic, dihomo-γ-linolenic) was calculated as the log-odds, log(*p*/(1 − *p*)), of the logistic regression model established in our previous study [24]: DI = −5.631 + 0.030 × (stearic acid) + 9.188 × (dihomo-γ-linolenic acid), where serum concentrations are expressed in µEq/L. Diagnostic performance was evaluated primarily at the cut-off value pre-specified in the previous study (0.4151) [24]. The cut-off value determined in the present cohort by Youden’s index (4.825) was used as a secondary analysis. The 95% CIs for sensitivity and specificity were calculated using the exact Clopper–Pearson method, and that for the AUC was calculated using Prism software. The associations between the DI (stearic, dihomo-γ-linolenic) level and tumor size, body mass index, and age were determined by Spearman’s rank correlation analysis.

Data management and statistical analyses were carried out using Prism software (version 9.0; GraphPad Software, La Jolla, CA, USA) and MetaboAnalyst 6.0. Significance levels were set at *p* < 0.05 for all tests. Data are presented as mean ± standard deviation.

## 3. Results

### 3.1. Validation of the CC Detection Method Using Serum FFAs in an Independent Cohort

**Figure 1:**
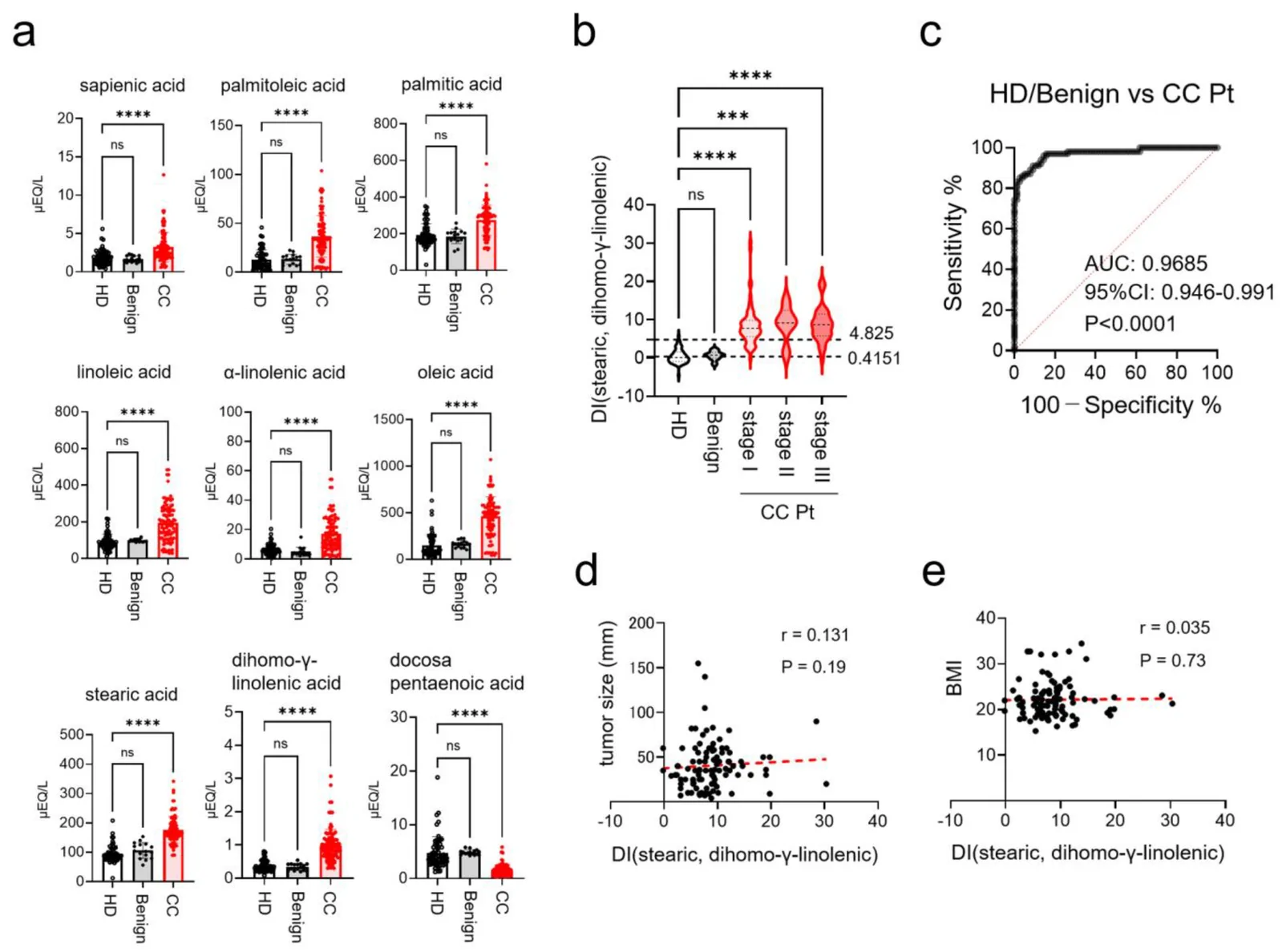
Performance and characterization of a diagnostic model for cervical cancer (CC) using serum free fatty acids (FFAs). (**a**) Levels of nine FFAs were measured by gas chromatography–mass spectrometry in serum samples from healthy donors (HD) (n = 60), patients with benign gynecological diseases (Benign) (n = 16), and patients with CC (n = 101) (Kruskal–Wallis test with Benjamini–Hochberg FDR correction across the nine FFAs, followed by Dunn’s multiple comparison test). (**b**,**c**) Performance of diagnostic index (DI) using stearic acid and dihomo-γ-linolenic acid (DI (stearic, dihomo-γ-linolenic)). (**b**) Comparison among HD, Benign, and stage I, II, and III CC (Kruskal–Wallis test followed by Dunn’s multiple comparison test). Dotted lines indicate the pre-specified cut-off value (0.4151) and the cut-off value determined in the present cohort (4.825). (**c**) Receiver operating characteristic curve analysis of HD and Benign versus patients with CC. (**d**,**e**) Characterization of DI (stearic, dihomo-γ-linolenic). Correlations between DI (stearic, dihomo-γ-linolenic) and tumor size (**d**) and body mass index (**e**) (Spearman’s rank correlation). ns: not significant; *** *p* < 0.001; **** *p* < 0.0001.

We previously demonstrated that nine FFAs were significantly altered in CC patients compared with healthy donors, and established a diagnostic model for CC using a combination of stearic acid and dihomo-γ-linolenic acid [24]. We first verified the use of this diagnostic model using serum FFA in an independent cohort (Table 1). In addition to healthy donors, we included patients with benign gynecological diseases (endometriosis, n = 9; uterine fibroids, n = 7), whose blood was drawn under the same perioperative conditions as the patients with CC. Eight FFAs, including sapienic, palmitoleic, palmitic, α-linolenic, linoleic, stearic, dihomo-γ-linolenic, and oleic acid, were elevated in patients with CC, while docosapentaenoic acid was decreased, compared with both healthy donors and patients with benign gynecological diseases; none of the nine FFAs differed between healthy donors and patients with benign gynecological diseases (Figure 1a). Furthermore, all nine FFAs demonstrated high diagnostic ability (Figure S1). We subsequently validated the diagnostic ability of the previously developed diagnostic model (DI; see Section 2.4) in this independent cohort. DI (stearic, dihomo-γ-linolenic) was higher in patients with CC than in healthy donors and patients with benign gynecological diseases, whereas it did not differ between the latter two groups (Figure 1b). The DI distinguished patients with CC from healthy donors and patients with benign gynecological diseases with an AUC of 0.969 (95% CI 0.946–0.991) (Figure 1c). At the cut-off value pre-specified in the previous study (0.4151), sensitivity was 97.0% (98/101), and specificity was 58.3% (35/60) in healthy donors and 31.3% (5/16) in patients with benign gynecological diseases (Figure 1b; Table 2). At the cut-off value determined in the present cohort (4.825), sensitivity was 82.2% (83/101), and specificity was 98.3% (59/60) in healthy donors and 100% (16/16) in patients with benign gynecological diseases (Figure 1b; Table 2). Because this cut-off value was derived from the data of healthy donors and patients with CC, the estimates for these groups are likely to be optimistic. The lower specificity at the pre-specified cut-off value was mainly attributable to higher dihomo-γ-linolenic acid levels in the non-cancer groups of the present cohort than in the healthy donors of the previous cohort (median 0.305 µEq/L in healthy donors and 0.348 µEq/L in patients with benign gynecological diseases vs. 0.097 µEq/L in the previous cohort) [24]. Furthermore, DI (stearic, dihomo-γ-linolenic) was independent of stage, tumor size, and pelvic lymph node metastasis, as factors associated with malignancy, supporting the results of previous studies [24] (Figure 1b,d and Figure S2). In addition, DI (stearic, dihomo-γ-linolenic) showed no correlation with body mass index, which is expected to affect serum FFA (Figure 1e), or with menopausal status and age, which are factors involved in metabolic changes (Figure S2). These results suggest that the DI can identify patients with CC across a range of clinical characteristics.

### 3.2. Serum-FFA Profiles and Expression of Fatty Acid Metabolism-Related Enzymes in hrHPV-Negative and hrHPV-Positive CC

**Figure 2:**
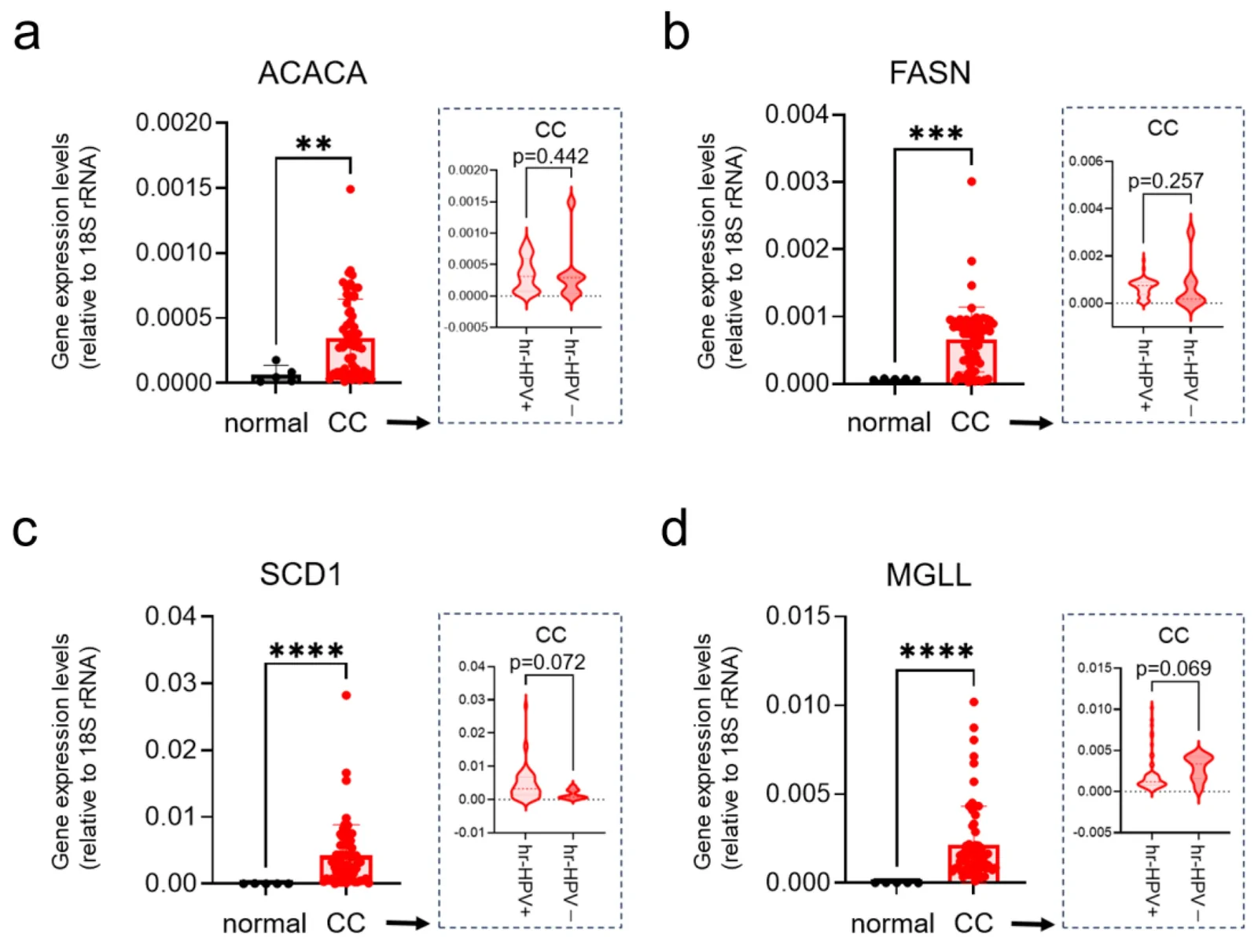
Gene expression of fatty acid metabolizing enzymes in high-risk human papillomavirus (hrHPV)-positive/negative cervical cancer (CC) and normal cervical tissues. (**a**–**d**) Expression levels of *ACACA* (**a**), *FASN* (**b**), *SCD1* (**c**), and *MGLL* (**d**) were measured by qPCR. Comparison between CC tissues (n = 64) and normal cervical tissues (n = 5) (**left panel**). Comparison between hrHPV-positive (n = 56) and hrHPV-negative (n = 8) CC (**right panel**); *p* values are shown. Mann–Whitney U test. ** *p* < 0.01, *** *p* < 0.001, **** *p* < 0.0001.

**Figure 3:**
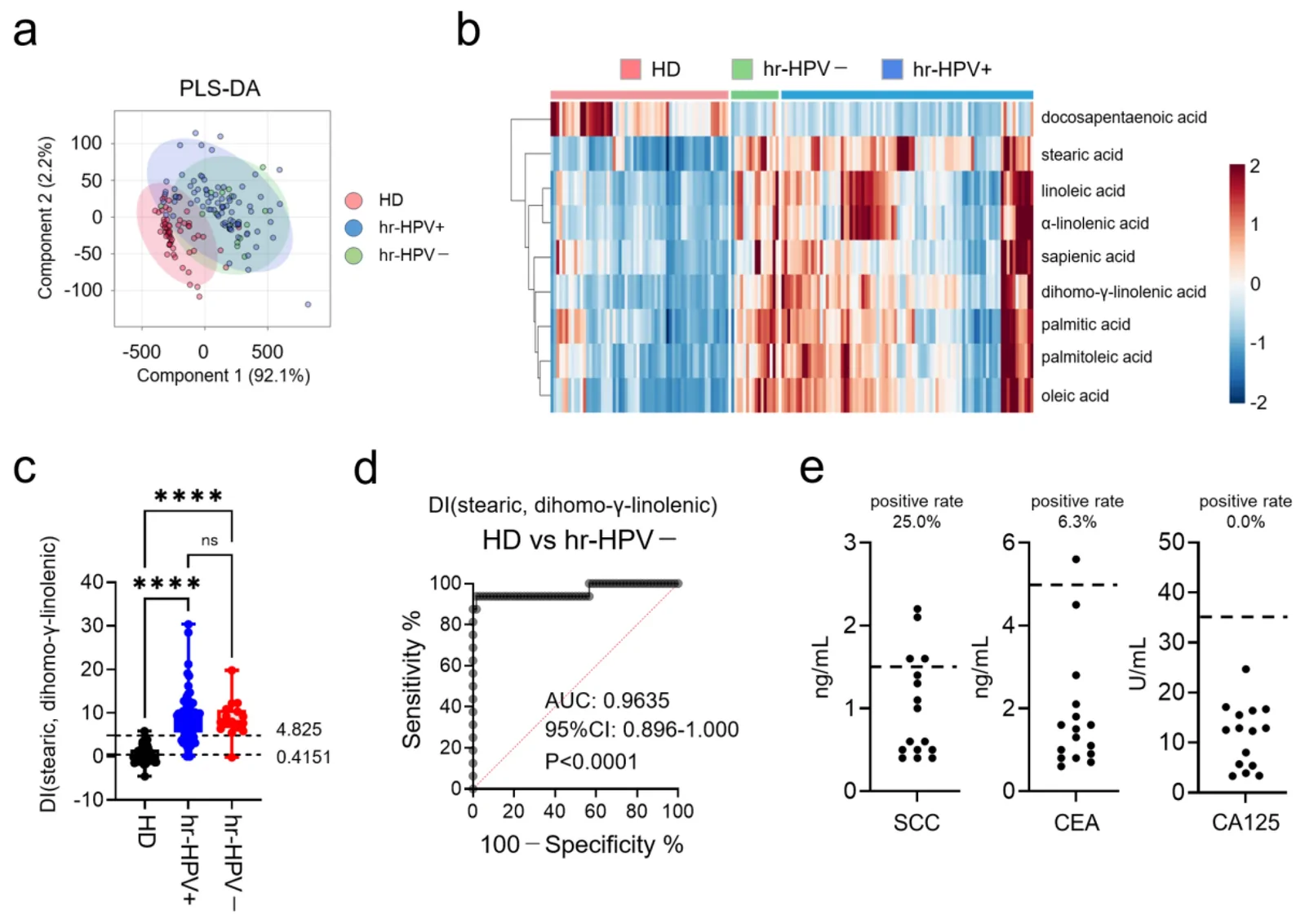
Serum free fatty acid (FFA) profiles and diagnostic index in high-risk human papillomavirus (hrHPV)-positive and hrHPV-negative cervical cancer (CC). (**a**–**d**) Levels of nine FFAs were measured by gas chromatography–mass spectrometry in serum samples from healthy donors (HD) (n = 60), patients with hrHPV-positive CC (n = 85), and patients with hrHPV-negative CC (n = 16). (**a**) Partial least squares discriminant analysis (PLS-DA) of serum-FFA profiles. (**b**) Heatmap of levels of nine FFAs in each sample. (**c**) Comparison of diagnostic index (DI) (stearic, dihomo-γ-linolenic) levels (Kruskal–Wallis test followed by Dunn’s multiple comparison test). Dotted lines indicate the pre-specified cut-off value (0.4151) and the cut-off value determined in the present cohort (4.825). (**d**) Receiver operating characteristic curve analysis of HD and patients with hrHPV-negative CC. (**e**) Tumor marker levels and positive rates in patients with hrHPV-negative CC. Dotted lines indicate cut-off values used in clinical practice. SCC: 1.5 ng/mL, CEA: 5.0 ng/mL, CA125: 35 U/mL. ns: not significant; **** *p* < 0.0001. SCC: squamous cell carcinoma antigen; CEA: carcinoembryonic antigen.

Current CC screening depends on hrHPV testing, and it is therefore important to develop highly sensitive diagnostic methods for detecting hrHPV-negative CC. We investigated if serum FFA could be used to detect hrHPV-negative CC. Changes in serum FFAs are known to be caused by altered expression of fatty acid metabolism enzymes in tumor tissues [23,24]. We therefore evaluated the expression of fatty acid metabolism-related enzymes in normal cervical tissue and CC tissue using qPCR, focusing on *ACACA*, *FASN*, and *SCD1*, which showed high expression in CC tissues based on open database analysis [24] and play central roles in fatty acid metabolism. Consistent with the database analysis, these enzymes were highly expressed in CC tissues compared with normal cervical tissues in the present cohort (Figure 2a–c). In addition, monoacylglycerol lipase (*MGLL*), which has been reported to control the composition of FFAs within cancer cells and promote cancer progression [26], was also highly expressed (Figure 2d). No significant differences in the expression of these enzymes were detected between hrHPV-negative and hrHPV-positive CC after FDR correction (Figure 2a–d). However, the effect sizes suggested lower expression of *SCD1* (fold change 0.36, 95% CI 0.15–1.10) and *FASN* (0.37, 0.14–1.27) and higher expression of *MGLL* (2.07, 0.95–3.58) in hrHPV-negative CC, although the number of hrHPV-negative samples was small (n = 8). We then determined whether serum FFAs differed in hrHPV-negative CC. PLS-DA and heatmap analysis revealed that the nine serum FFAs showed similar patterns in hrHPV-negative and hrHPV-positive CC, which differed from those in healthy donors (Figure 3a,b and Figure S3). No significant differences in the nine FFAs were detected between hrHPV-negative and hrHPV-positive CC after FDR correction, and the effect sizes were small (Table 3). For the two components of the DI, the fold changes (hrHPV-negative/hrHPV-positive) were 1.05 (95% CI 0.91–1.18) for stearic acid and 1.02 (0.82–1.27) for dihomo-γ-linolenic acid.

### 3.3. DI (Stearic, Dihomo-γ-Linolenic) Detected hrHPV-Negative CC

We next evaluated the ability of DI (stearic, dihomo-γ-linolenic) to detect hrHPV-negative CC. The DI did not differ between hrHPV-negative and hrHPV-positive CC (adjusted *p* > 0.9999; difference 0.25, 95% CI −1.61 to 2.42), and both groups showed higher DI values than healthy donors (adjusted *p* < 0.0001 for both) (Figure 3c). The DI detected 15 of 16 patients with hrHPV-negative CC (93.8%; 95% CI 69.8–99.8%) at both the pre-specified and the re-derived cut-off values (Figure 3c; Table 2), and the AUC for distinguishing hrHPV-negative CC from healthy donors was 0.964 (95% CI 0.896–1.000) (Figure 3d). Because all hrHPV-negative cases were adenocarcinomas (gastric type, n = 12; clear cell type, n = 4), we also compared hrHPV-negative adenocarcinoma (n = 16) with hrHPV-positive adenocarcinoma (n = 22). The DI did not differ between these groups (median 7.94 vs. 6.80; difference 1.47, 95% CI −0.56 to 4.01; *p* = 0.198), and 15/16 and 21/22 cases, respectively, were positive at the pre-specified cut-off value. In contrast, the positivity rates for the commonly used tumor markers squamous cell carcinoma antigen (SCC), carcinoembryonic antigen (CEA), and CA125 in hrHPV-negative CC were 25.0%, 6.3%, and 0.0%, respectively (Figure 3e). These results suggest that the detection ability of the DI is maintained in hrHPV-negative CC.

## 4. Discussion

This study demonstrated that the serum-FFA-based DI established in our previous study, based on stearic acid and dihomo-γ-linolenic acid, detected CC in an independent cohort, including hrHPV-negative cases. Serum-FFA profiles were similar in hrHPV-negative and hrHPV-positive CC, suggesting that alterations in serum-FFA levels in CC may not depend on HPV infection status. This approach addresses a gap in current CC screening strategies, which predominantly rely on hrHPV testing and may thus miss around 5–10% of CC cases, which are hrHPV-negative. The diagnostic performance of the DI for hrHPV-negative CC, particularly compared with conventional tumor markers (SCC: 25.0%, CEA: 6.3%, CA125: 0.0%), suggests that serum-FFA profiling could complement HPV-based testing.

The altered fatty acid profiles in patients with CC are thought to reflect the comprehensive survival strategy of cancer cells. Increased levels of monounsaturated fatty acids, including palmitoleic and oleic acid, are linked to the activation of SCD1, suggesting that cancer cells may mitigate endoplasmic reticulum stress-dependent apoptosis and lipotoxicity [27,28]. Furthermore, the increase in n-6 polyunsaturated fatty acids such as linoleic and dihomo-γ-linolenic acid is thought to be involved in the activation of inflammatory and proliferative signals via the arachidonic acid cascade [29]. Notably, elevated dihomo-γ-linolenic acid levels may create a microenvironment that promotes cancer proliferation through abnormal production of prostaglandin E1 and the regulation of cyclooxygenase and lipoxygenase signaling [30,31]. In contrast, docosapentaenoic acid, which was decreased in patients with CC, has demonstrated anticancer effects in human colorectal cancer cell line xenograft mouse models through reduced expression of cyclooxygenase-2, cyclin D1, c-myc, Bcl-2, matrix metalloproteinase-9, and vascular endothelial growth factor proteins, as well as enhanced apoptosis [32]. These findings therefore suggest that CC cells establish a fatty acid metabolic environment favorable to their survival, and that similar fatty acid profile changes are observed in hrHPV-negative and hrHPV-positive CC. Stearic acid, however, has previously been reported to have anticancer properties [33], and its increased levels in the current study appear to be inversely correlated with cancer cell survival strategies. Elevated stearic acid levels have not been confirmed in colorectal, lung, or ovarian cancers [22,23], suggesting that this phenomenon may be specific to CC. The mechanistic basis for this paradoxical stearic acid elevation remains to be elucidated and represents a critical area for future research to optimize the diagnostic application of this biomarker.

The upregulation of fatty acid metabolism-related enzymes, particularly MGLL and SCD1, in CC tissues may provide insights into CC metabolic reprogramming. MGLL catalyzes the hydrolysis of monoacylglycerols to generate FFAs and glycerol, serving as a critical regulator of fatty acid homeostasis in cancer cells [26]. Elevated MGLL expression may contribute to the altered serum-FFA profiles observed in our study, given that this enzyme controls the release of bioactive fatty acids that can promote tumor growth and survival through multiple pathways, including prostaglandin synthesis and membrane lipid composition [34,35,36]. Similarly, SCD1 catalyzes the conversion of saturated fatty acids to monounsaturated fatty acids, which is essential for maintaining membrane fluidity and protecting cells from lipotoxicity-induced apoptosis [28,37]. Increased SCD1 expression in CC tissues corresponds with the elevated levels of monounsaturated fatty acids detected in patient serum, suggesting that this metabolic adaptation may represent a survival mechanism in CC cells. Lipid metabolic reprogramming has been implicated in CC, and HPV infection has been reported to affect host lipid metabolism [19,20,38]; MGLL has also been shown to promote CC progression [39]. However, direct evidence comparing lipolysis and de novo fatty acid synthesis between hrHPV-negative and hrHPV-positive CC is lacking. In the present study, no significant differences in the expression of these enzymes were detected between hrHPV-negative and hrHPV-positive CC, although the effect sizes suggested lower SCD1 and FASN expression and higher MGLL expression in hrHPV-negative CC. We therefore hypothesize that hrHPV-negative CC may rely relatively more on lipolysis than on de novo fatty acid synthesis, so that different metabolic routes could lead to similar changes in serum FFAs. Because the number of hrHPV-negative tissue samples was small and enzyme activities and metabolic fluxes were not measured, this hypothesis requires validation in future studies.

In this independent cohort, the discriminative ability of the DI was reproduced, but specificity at the cut-off value pre-specified in our previous study was lower than previously reported [24]. This was mainly attributable to higher dihomo-γ-linolenic acid levels in the non-cancer groups of the present cohort. Because the DI assigns a large coefficient to dihomo-γ-linolenic acid, which is present at low concentrations in individuals without cancer, small differences in its measured levels can substantially shift DI values. For clinical application, the absolute threshold will therefore need to be calibrated, for example by using a stable isotope-labelled internal standard for dihomo-γ-linolenic acid and common quality-control materials across batches and sites. Diagnostic models that are more robust to calibration differences, such as those based on log-transformed concentrations or within-sample fatty acid ratios, or those that include fatty acids present at higher concentrations, may also be useful.

The clinical implications of our findings are particularly relevant for improving CC screening programs. Current HPV-based screening strategies are effective in most cases, but have an inherent limitation for detecting hrHPV-negative CC, which accounts for approximately 5–10% of all cases, depending on the population, testing method, and diagnostic criteria [40,41]. These cases include truly HPV-independent tumors, such as gastric-type adenocarcinoma, as well as false-negative results caused by low viral load, sampling problems, or assay limitations [40,41]. With the widespread adoption of HPV vaccines, the incidence of HPV-related CC is gradually declining, while the relative incidence of HPV-unrelated CC is showing an upward trend [42]. This epidemiological shift highlights the need to develop HPV-independent diagnostic methods. Because the clinically relevant population consists of women with negative HPV test results, regardless of their true viral status, a marker that detects CC irrespective of the HPV test result could help reduce missed diagnoses. DI (stearic, dihomo-γ-linolenic) showed a higher positivity rate for hrHPV-negative cases (93.8%) than conventional tumor markers (SCC, CEA, CA125). The use of serum-FFA profiling as a complementary tool could help reduce the number of missed diagnoses, particularly of aggressive hrHPV-negative tumors that often present with a poorer prognosis. Moreover, the blood-based nature of this test has practical advantages, including minimal invasiveness and the potential for integration into routine health checkups. Future studies in screening populations are needed to evaluate whether FFA profiling could be used, for example, as a triage tool for women with inconclusive HPV or cytology results.

This study has several limitations. First, this was a case–control study, reported according to the STARD 2015 guidelines (Table S2 and Figure S4), with a relatively small number of participants, particularly patients with hrHPV-negative CC (16 serum and 8 tissue samples) and benign gynecological diseases, and no formal sample size calculation was performed; the specificity of the DI against CIN, other gynecological cancers, and metabolic conditions should be examined in larger cohorts, including screening populations. Second, hrHPV status was determined using cytology specimens without confirmation by p16 immunohistochemistry or tissue-based HPV testing, and histological subtypes were not centrally reviewed. Third, detailed pre-analytical information, such as fasting status, was not available, and the healthy donors were somewhat younger than the patients, with no clinical information other than age; although patients with benign gynecological diseases sampled under the same perioperative conditions showed DI values similar to those of healthy donors, residual effects of sampling conditions cannot be fully excluded. In addition, because serum samples from patients with benign gynecological diseases were analyzed in a separate batch, batch effects cannot be excluded in comparisons involving this group. Finally, tissue composition, such as cellularity, stromal content, and necrosis, was not assessed; differences between normal and tumor tissues may therefore have contributed to the observed differences in gene expression, and the comparison between hrHPV-negative and hrHPV-positive CC, which was made among tumor tissues, may have also been affected by variation in tumor content.

Despite these limitations, our findings indicate that the detection ability of the serum-FFA-based DI is maintained in hrHPV-negative CC in an independent cohort. As HPV vaccination becomes more widespread and the relative proportion of HPV-independent CC increases, blood-based metabolic markers that do not rely on viral detection may become increasingly valuable in CC detection.

## 5. Conclusions

In conclusion, this study suggests that a serum-FFA-based diagnostic model can detect CC in an independent cohort, including hrHPV-negative cases, which may be missed by current HPV-based testing. This diagnostic approach may complement current HPV test-based screening. Further validation, including calibration of the cut-off value and evaluation in screening populations, is warranted before this serum-FFA diagnostic approach can be incorporated into CC screening programs.

## Figures and Tables

**Table 1:** Characteristics of participants whose serum free fatty acid concentrations were measured in this study.

Characteristic	Healthy Donors (n = 60)	Benign Gynecological Diseases (n = 16)	Patients with CC (n = 101)	
			hrHPV-Positive (n = 85)	hrHPV-Negative (n = 16)
Age, years				
Median (range)	40.5 (21–64)	42.0 (30–74)	46.0 (28–77)	46.5 (30–62)
Menopausal status				
Pre/post	–	12/4	51/34	11/5
BMI				
Median (range)	–	22.1 (15.9–31.1)	21.5 (15.3–33.5)	22.6 (16.5–28.3)
Tumor size, mm				
Median (range)	–	–	35 (6–155)	40 (7–82)
Histopathological subtypes				
Squamous cell carcinoma	–	–	63 (74.1%)	0 (0.0%)
Adenocarcinoma	–	–	22 (25.9%)	16 (100.0%)
FIGO 2018 stage				
I (IA/IB)	–	–	53 (9/44)	11 (3/8)
II (IIA/IIB)	–	–	5 (2/3)	0 (0/0)
III (IIIA/IIIB/IIIC)	–	–	27 (0/2/25)	5 (0/0/5)
IV (IVA/IVB)	–	–	0 (0/0)	0 (0/0)

CC: cervical cancer; hrHPV: high-risk HPV; BMI: body mass index; FIGO: International Federation of Gynecology and Obstetrics.

**Table 2:** Diagnostic performance of the DI (stearic, dihomo-γ-linolenic).

	DI (Stearic, Dihomo-γ-Linolenic)	
	Pre-Specified Cut-Off 0.4151 (Primary)	Re-Derived Cut-Off 4.825 (Secondary)
Sensitivity		
All CC (n = 101)	97.0% (91.6–99.4); 98/101	82.2% (73.3–89.1); 83/101
hrHPV-positive CC (n = 85)	97.6% (91.8–99.7); 83/85	80.0% (69.9–87.9); 68/85
hrHPV-negative CC (n = 16)	93.8% (69.8–99.8); 15/16	93.8% (69.8–99.8); 15/16
Specificity		
Healthy donors (n = 60)	58.3% (44.9–70.9); 35/60	98.3% (91.1–100.0); 59/60
Benign gynecological diseases (n = 16)	31.3% (11.0–58.7); 5/16	100% (79.4–100.0); 16/16

Values are % (exact 95% CI); number of correctly classified participants/total number in each group. The pre-specified cut-off was taken from our previous study [24]. The re-derived cut-off was determined in the present cohort by Youden’s index using healthy donors and patients with CC, and is likely to give optimistic estimates for these groups. DI: diagnostic index; CC: cervical cancer; hrHPV: high-risk human papillomavirus.

**Table 3:** Serum free fatty acid levels and effect sizes in hrHPV-positive and hrHPV-negative cervical cancer.

Free Fatty Acid	hrHPV-Positive CC (n = 85)	hrHPV-Negative CC (n = 16)	Fold Change (95% CI)	*p* Value	FDR-Adjusted *p*
Sapienic acid	3.20 ± 1.91	3.33 ± 1.97	1.03 (0.80–1.34)	0.849	0.930
Palmitoleic acid	36.4 ± 21.8	38.0 ± 18.9	1.04 (0.78–1.47)	0.791	0.930
Palmitic acid	268.4 ± 83.7	303.0 ± 65.1	1.15 (1.00–1.33)	0.043	0.388
Linoleic acid	191.1 ± 103.3	210.1 ± 96.0	1.13 (0.85–1.55)	0.421	0.741
α-Linolenic acid	16.8 ± 11.5	19.0 ± 12.1	1.19 (0.79–1.84)	0.432	0.741
Oleic acid	451.0 ± 210.3	518.4 ± 180.5	1.15 (0.96–1.43)	0.146	0.655
Stearic acid	175.7 ± 46.1	178.5 ± 41.1	1.05 (0.91–1.18)	0.488	0.741
Dihomo-γ-linolenic acid	0.98 ± 0.49	0.98 ± 0.43	1.02 (0.82–1.27)	0.930	0.930
Docosapentaenoic acid	1.56 ± 0.99	1.71 ± 1.03	1.11 (0.84–1.50)	0.494	0.741

Concentrations (µEq/L) are presented as mean ± standard deviation. Fold changes (hrHPV-negative/hrHPV-positive) are Hodges–Lehmann estimates calculated on log2-transformed concentrations. *p* values were obtained by the Mann–Whitney U test and adjusted using the Benjamini–Hochberg FDR method. CC: cervical cancer; hrHPV: high-risk human papillomavirus.

## Data Availability

The data presented in this study are available on request from the corresponding author. The data are not publicly available due to restrictions in the ethical approval.

## Supplementary Materials

The following supporting information can be downloaded at https://www.mdpi.com/article/10.3390/diagnostics16203387/s1. Figure S1. Serum levels of nine free fatty acids were useful diagnostic markers for patients with cervical cancer. Figure S2. Diagnostic index (DI) (stearic, dihomo-γ-linolenic) was unaffected by clinical parameters in patients with cervical cancer (CC). Figure S3. Serum free fatty acid (FFA) profiles in high-risk human papillomavirus (hrHPV)-positive and hrHPV-negative cervical cancer (related to Figure 3b). Figure S4. STARD flow diagram of participants. Table S1. Gas chromatography–mass spectrometry analyses of serum free fatty acids; Table S2. STARD 2015 checklist.

## Abbreviations

The following abbreviations are used in this manuscript:

ACACA	Acetyl-CoA carboxylase 1
AUC	Area under the curve
CC	Cervical cancer
CEA	Carcinoembryonic antigen
CI	Confidence interval
DI	Diagnostic index
FASN	Fatty acid synthase
FDR	False discovery rate
FFA	Free fatty acid
FIGO	The International Federation of Gynecology and Obstetrics
GC-MS	Gas chromatography–mass spectrometry
HPV	Human papillomavirus
hrHPV	High-risk human papillomavirus
MGLL	Monoacylglycerol lipase
PLS-DA	Partial least squares discriminant analysis
SCC	Squamous cell carcinoma antigen
SCD1	Stearoyl-CoA desaturase 1
STARD	Standards for Reporting of Diagnostic Accuracy Studies
