# Supplementary material for: Serum Free Fatty Acid-Based Diagnostic Index for Cervical Cancer Including High-Risk HPV-Negative Cases: An Independent Validation Study
Source: Diagnostics (Basel). 2026 Oct 18;16(20):3387. doi: 10.3390/diagnostics16203387 (PMC13703187; doi:10.3390/diagnostics16203387)
Supplement: Supplementary file 1 [file diagnostics-16-03387-s001.zip › diagnostics-4621154-supplemental materials.pdf]

Supplementary Material

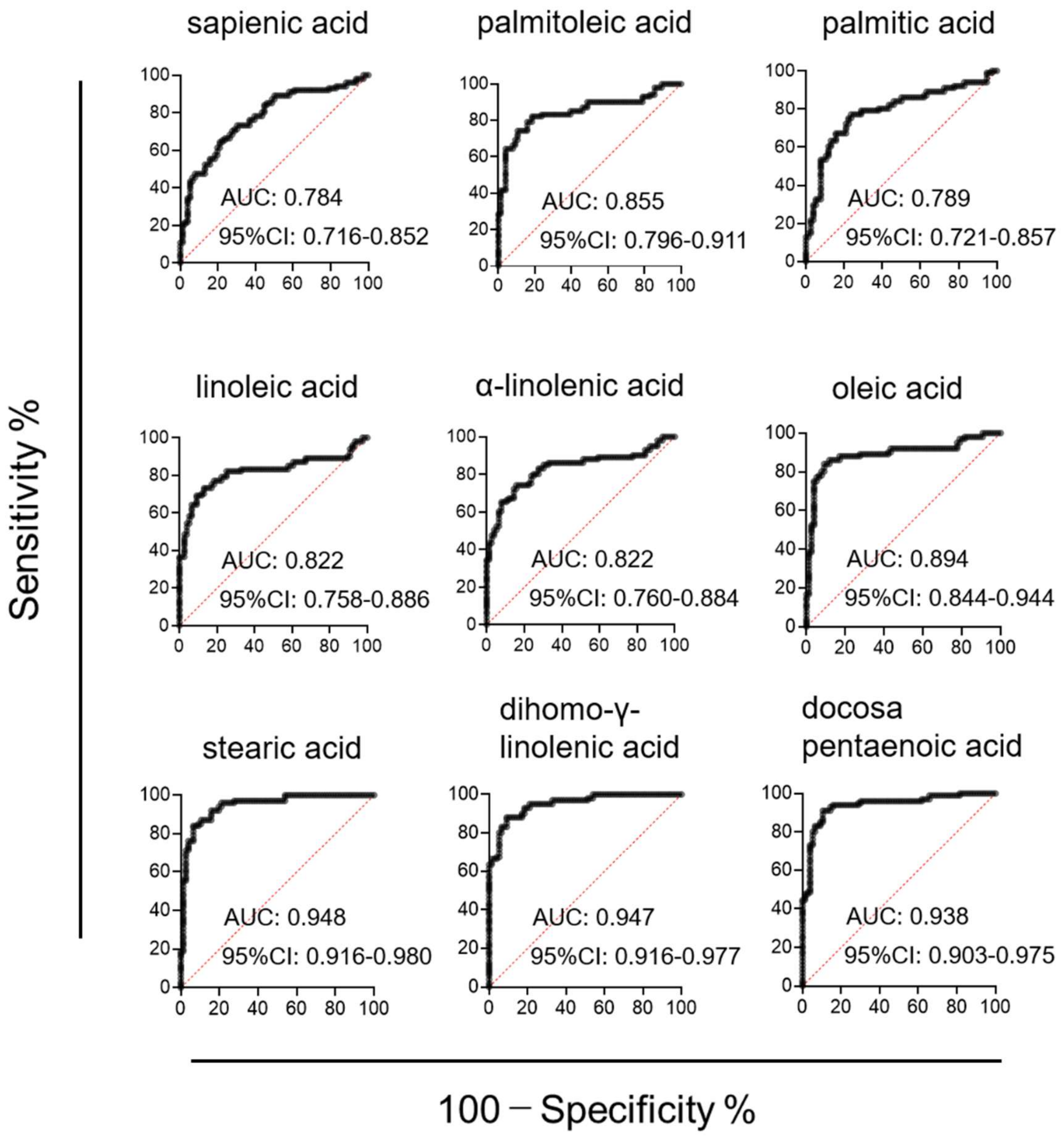

Figure S1. Serum levels of nine free fatty acids were useful diagnostic markers for patients with cervical cancer. Receiver operating characteristic curves for distinguishing patients with cervical cancer (n = 101) from healthy donors and patients with benign gynecological diseases (n = 76) using the nine serum free fatty acids shown in Figure 1a. AUC: area under the curve; CI: confidence interval.

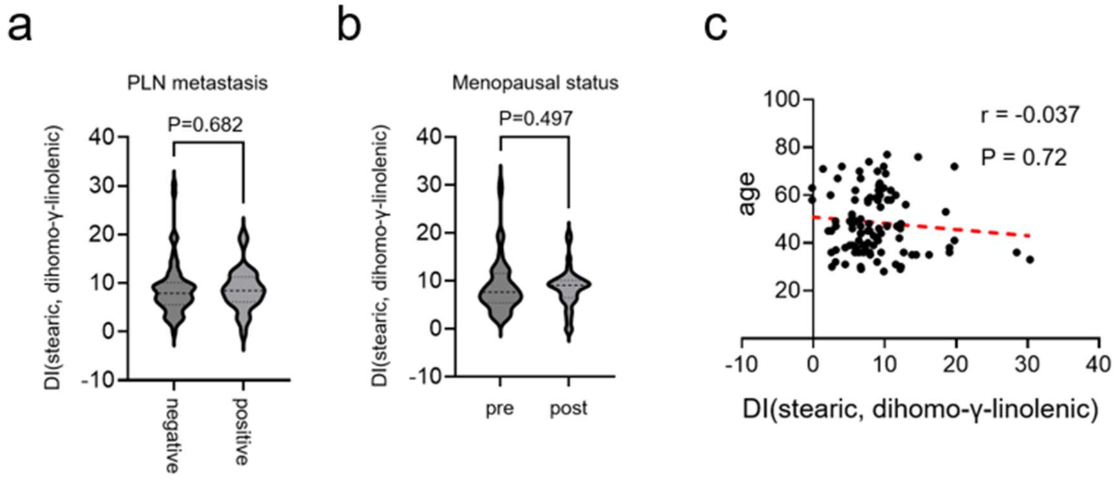

Figure S2. Diagnostic index (DI) (stearic, dihomo-γ-linolenic) was unaffected by clinical parameters in patients with cervical cancer (CC). **(a)** Comparison of DI (stearic, dihomo-γ-linolenic) in patients with pelvic lymph node (PLN) metastasis-negative and -positive CC. **(b)** Comparison of DI (stearic, dihomo-γ-linolenic) in premenopausal and postmenopausal patients with CC. **(a, b)** Mann–Whitney U test. **(c)** Correlation between DI (stearic, dihomo-γ-linolenic) and age (Spearman's rank correlation).

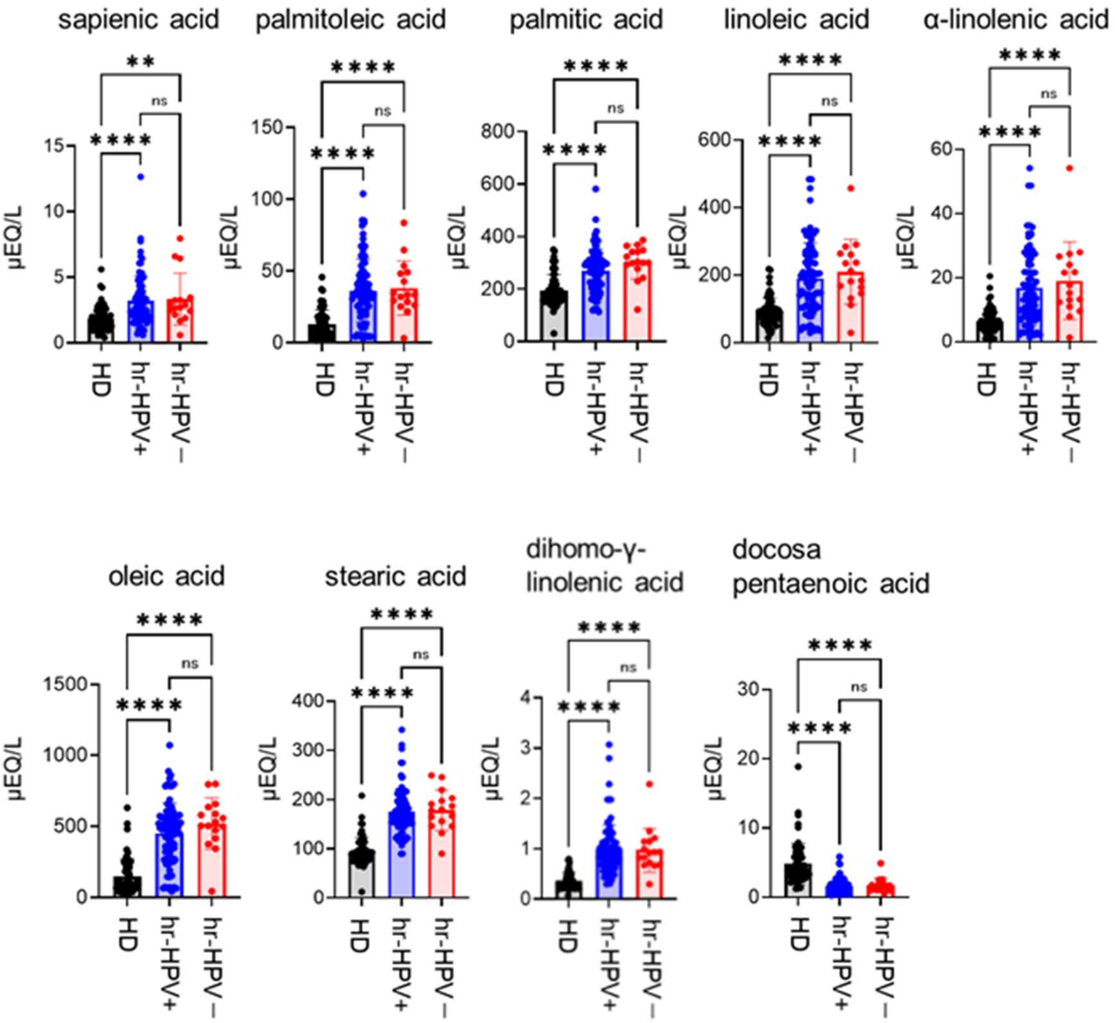

Figure S3. Serum free fatty acid (FFA) profiles in high-risk human papillomavirus (hrHPV)-positive and hrHPV-negative cervical cancer (CC) (related to Figure 3b). Levels of nine FFAs were measured by gas chromatography-mass spectrometry in serum samples from healthy donors (HD) (n = 60), patients with hrHPV-positive CC (n = 85), and patients with hrHPV-negative CC (n = 16). Comparisons for each FFA are shown (Kruskal–Wallis test followed by Dunn's multiple comparison test). **P < 0.01, ****P < 0.0001. ns: not significant.

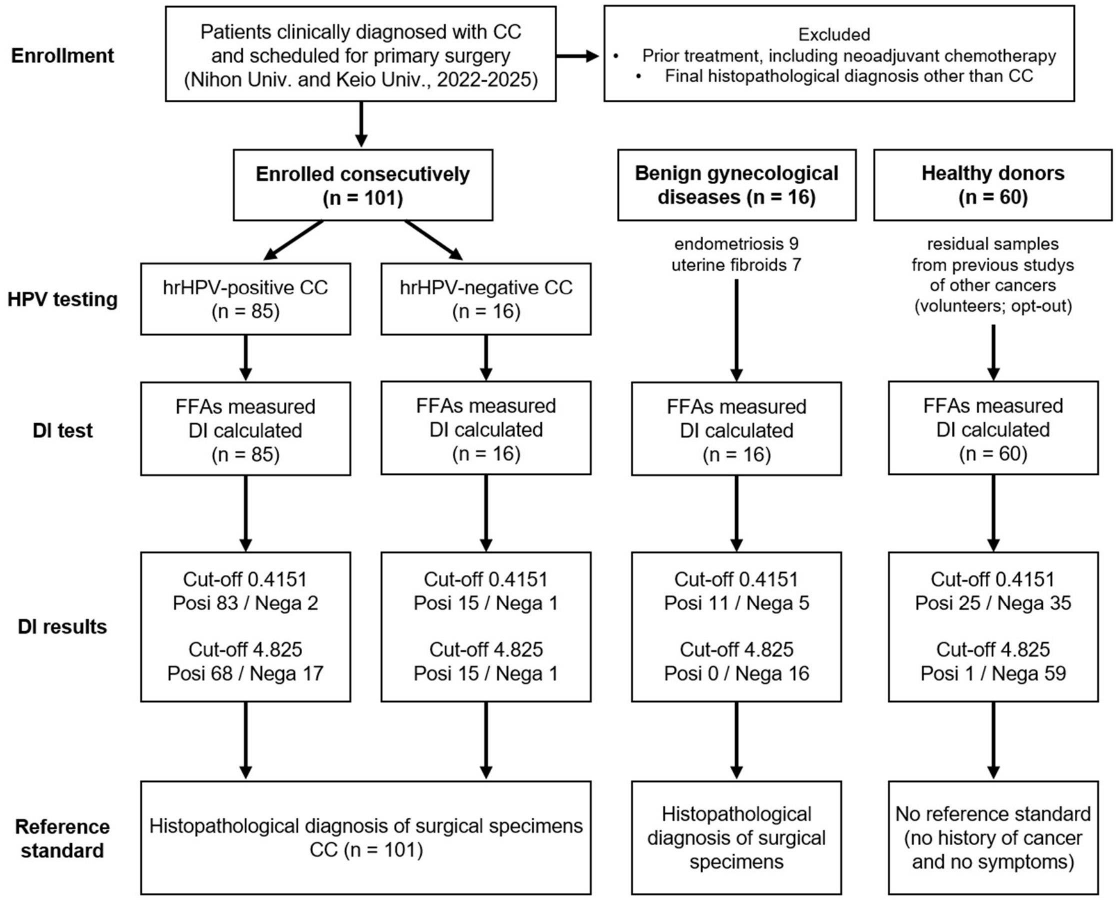

Figure S4. STARD flow diagram of participants. CC, cervical cancer; DI, diagnostic index (stearic, dihomo-γ-linolenic); FFA, free fatty acid; hrHPV, high-risk human papillomavirus. The cut-off value of 0.4151 was pre-specified in our previous study [24]; the cut-off value of 4.825 was determined in the present cohort by Youden's index.
